# Lumbar Hernia: A Report of a Unique Case and Review of the Literature

**DOI:** 10.7759/cureus.110253

**Published:** 2026-06-04

**Authors:** Ethan Gipsman, Dvir Froylich

**Affiliations:** 1 Orthopedics, Rabin Medical Center, Beilinson Hospital, Petah Tikva, ISR; 2 General Surgery, Bariatric Service, Carmel Medical Center, Haifa, ISR

**Keywords:** aesthetic abdominoplasty, grynfeltt hernia, grynfeltt–lesshaft triangle, hernia, lumbar hernia repair, petit's hernia, petit triangle, primary lumbar hernia, traumatic hernia

## Abstract

We present a case of a 53-year-old female with a left-sided superior lumbar hernia who underwent successful laparoscopic reduction and mesh repair. The case report is accompanied by an extensive literature review of lumbar hernias. The review’s purpose is to provide details of what can be expected when treating this rare defect. Publications relevant to the reporting of lumbar hernias were found using the National Library of Medicine database. Each case report was cataloged and reviewed. Repair type (open vs. laparoscopic), patient age, gender, presenting symptoms, complications, hernia site, length of hospital stay, and trauma/surgical history were recorded in a spreadsheet.

Of the 41 patients, 11 had complications following hernia repair, the most common being recurrence, which occurred in seven patients. Of the 11 patients who reported complications, four had an open procedure, and eight had a laparoscopy (one patient underwent both open and laparoscopic surgery and had complications after both procedures). In two of the seven cases of recurrence (28.5%), an open surgical approach was used for the revision surgery, where the initial repair had been attempted laparoscopically. Our review found that the most common causes of traumatic injury in patients who went on to develop a lumbar hernia were motor vehicle accidents (29.2%) or a bone graft taken from the iliac crest (14.6%). Our review also found that the patient in our case had a unique surgical history (abdominoplasty, breast implants, implant removal) amongst lumbar hernia patients.

Our literature review provides a clinical picture of the presentation of a lumbar hernia patient with a unique surgical history, as well as the most statistically relevant history. It can be speculated that our patient’s unique surgical history may have contributed to the development of a lumbar hernia, although it cannot be deemed causation due to it being a unique case.

While lumbar hernias are an extremely rare occurrence, they should be considered in patients with a mass accompanied by flank pain, especially in patients with a history of trauma or iliac crest bone grafting. Laparoscopic repair appears to offer a shorter hospital stay, but recurrence remains a notable complication, possibly due to the unique nature of lumbar hernias and surgeons’ inexperience in treating them. When deciding on treatment, the surgeon should take into account the site of the hernia and their experience in laparoscopic hernia repair, and ultimately make the decision together with the patient.

## Introduction

Lumbar hernias may be congenital or acquired, often following abdominal trauma or surgery to the corresponding flank. Lumbar hernias are extremely rare, with approximately 300 cases reported over the last three centuries [1]. Lumbar hernias can be particularly difficult to diagnose as the symptoms are vague and non-specific (unlocalized back pain). The main presentation is a lump in the flank that may increase with activity or disappear when lying down [2]. Diagnosis can be further complicated in an obese or postoperative patient, as the hernia can easily be mistaken for a lipoma or general postoperative pain. Complications of this hernia are rare, as lumbar hernias are generally not prone to incarceration [3].

The first of two potential locations for lumbar hernias, i.e., the superior lumbar triangle, is an inverted triangle consisting of three borders: superiorly, the lower border of the 12th rib; medially, the erector spinae and quadratus lumborum muscles; and laterally, the internal oblique muscle. This is also known as Grynfeltt’s hernia. The second potential location for a lumbar hernia is the inferior lumbar triangle. This consists of the iliac crest as the inferior border, the latissimus dorsi muscle medially, and the external oblique muscle on the lateral side [4]. This hernia is also referred to as Petit’s hernia. Weakness of the lumbodorsal fascia in these areas leads to the projection of extraperitoneal fat through the triangle and results in a hernia sac. Repair of a lumbar hernia, like most hernias, is generally performed using a prosthetic mesh in either an open or laparoscopic approach. This report will discuss a case of a superior lumbar hernia witnessed by the authors, as well as a review of the literature on lumbar hernias.

## Case presentation

**Figure 1:**
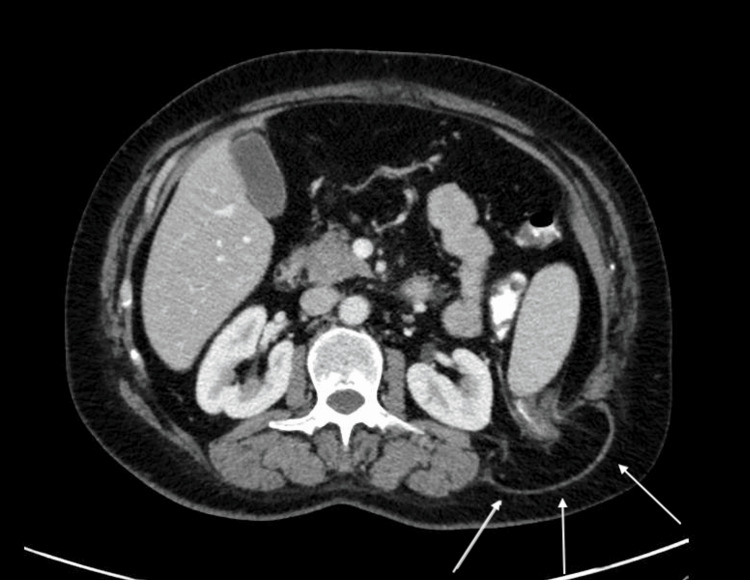
CT axial view. The arrows indicate the lumbar hernia defect.

**Figure 2:**
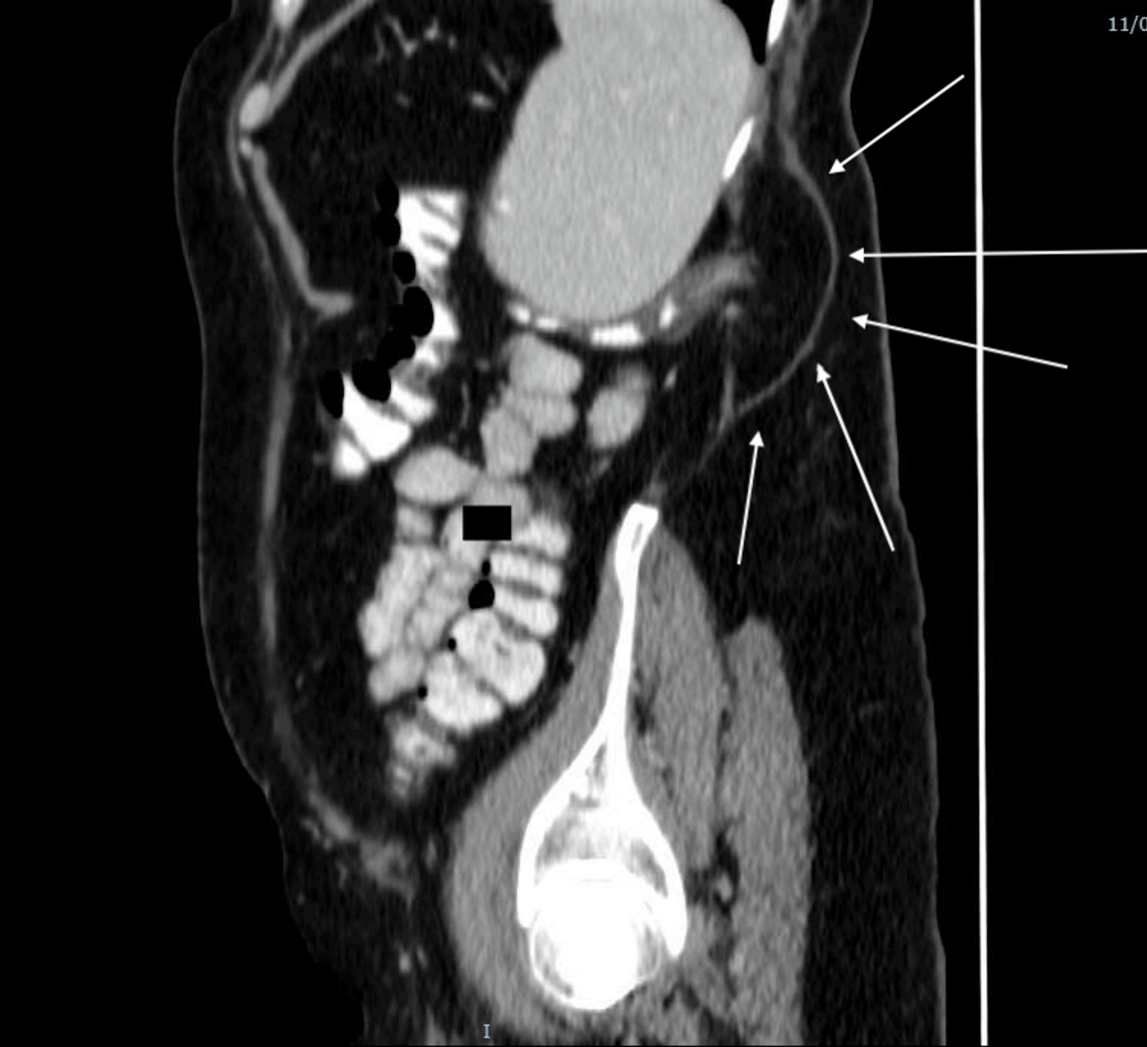
CT sagittal view. The arrows indicate the lumbar hernia defect.

A 53-year-old female presented to the Carmel Medical Center Clinic in Haifa, Israel, with intermittent stabbing pain and fullness in her left lower back for the past three years. The pain was not relieved or worsened by any specific activity, although the pain has increased over the past year, which ultimately led her to seek treatment. She had no relevant medical or family history. Her past surgical history included breast implants in 2000, an abdominoplasty in 2008, and removal of her breast implants in 2020. The patient was referred to the clinic due to a suspected lipoma. She presented with a soft, nontender, palpable mass measuring 6 cm. The mass was reducible upon palpation and was therefore treated as a suspected hernia (a lipoma would not be reducible). The patient was referred to a computed tomography (CT) scan. Upon CT scan, a lumbar hernia sac measuring 25 x 65 millimeters, composed of fat emerging through an opening of approximately 15 millimeters in size, was demonstrated on the left posterior aspect of the abdomen (Figures 1, 2). After discussing the findings and treatment options, the patient elected to undergo surgery to repair the lumbar hernia, and she was scheduled for a laparoscopic approach repair.

**Figure 3:**
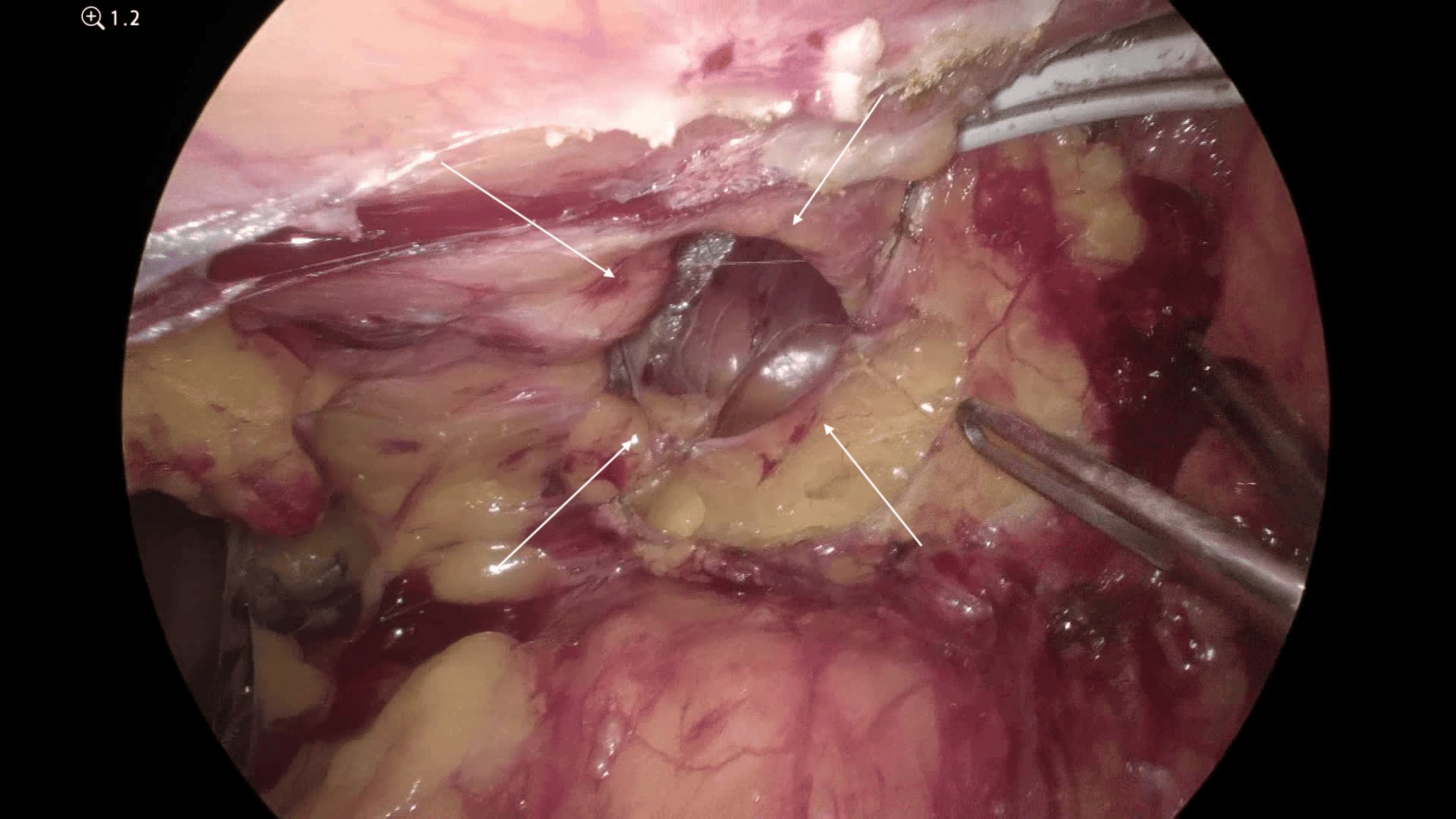
Identifying the defect intraoperatively. The white arrows point to the lumbar hernia defect.

**Figure 4:**
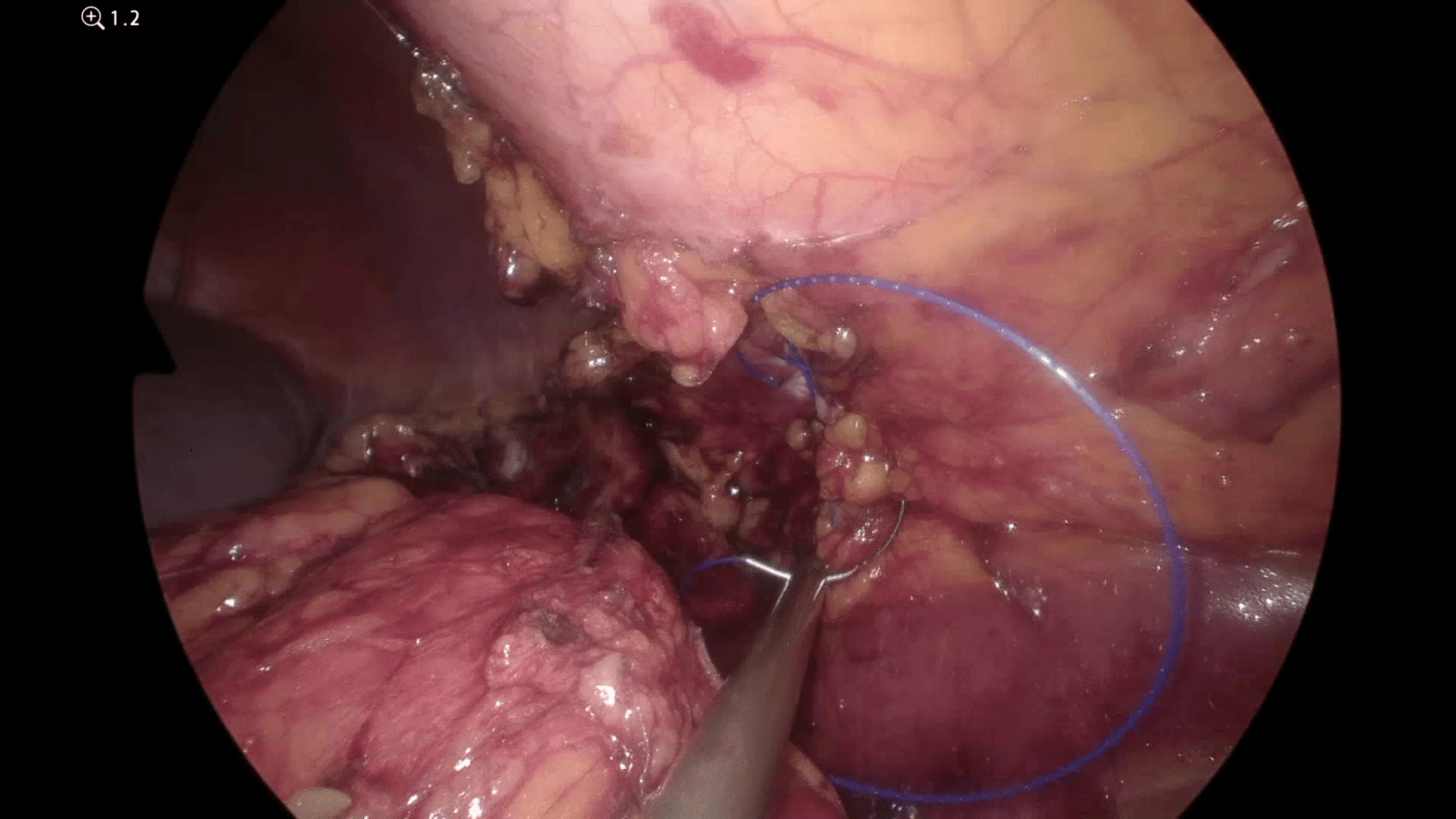
Reducing the defect. The defect was closed using a non-absorbable 0 running V-Loc suture (Medtronic).

**Figure 5:**
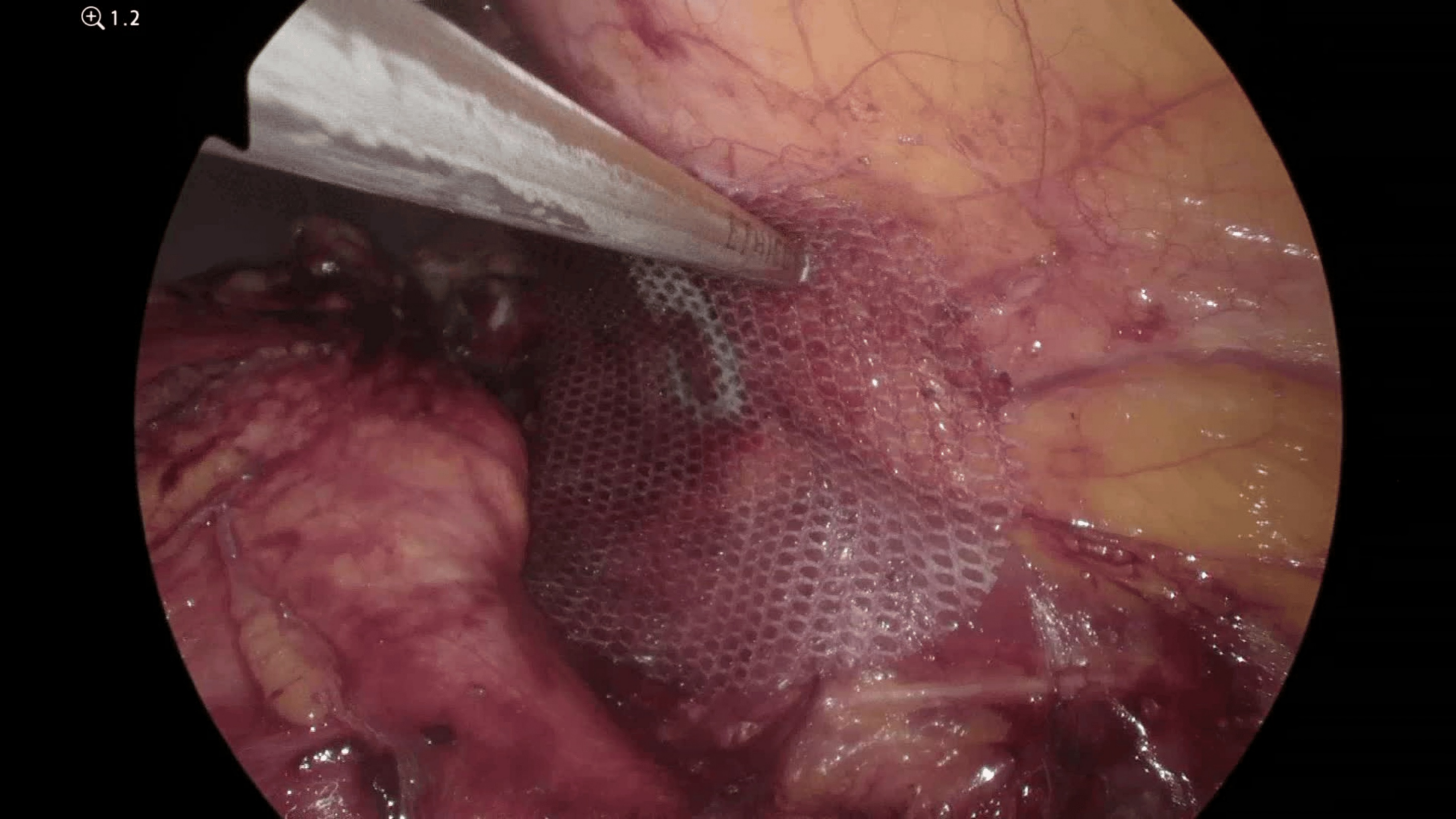
Deploying Symbotex mesh (Medtronic) over the closed defect. A Symbotex mesh of 10 cm in diameter was deployed over the reduced defect.

**Figure 6:**
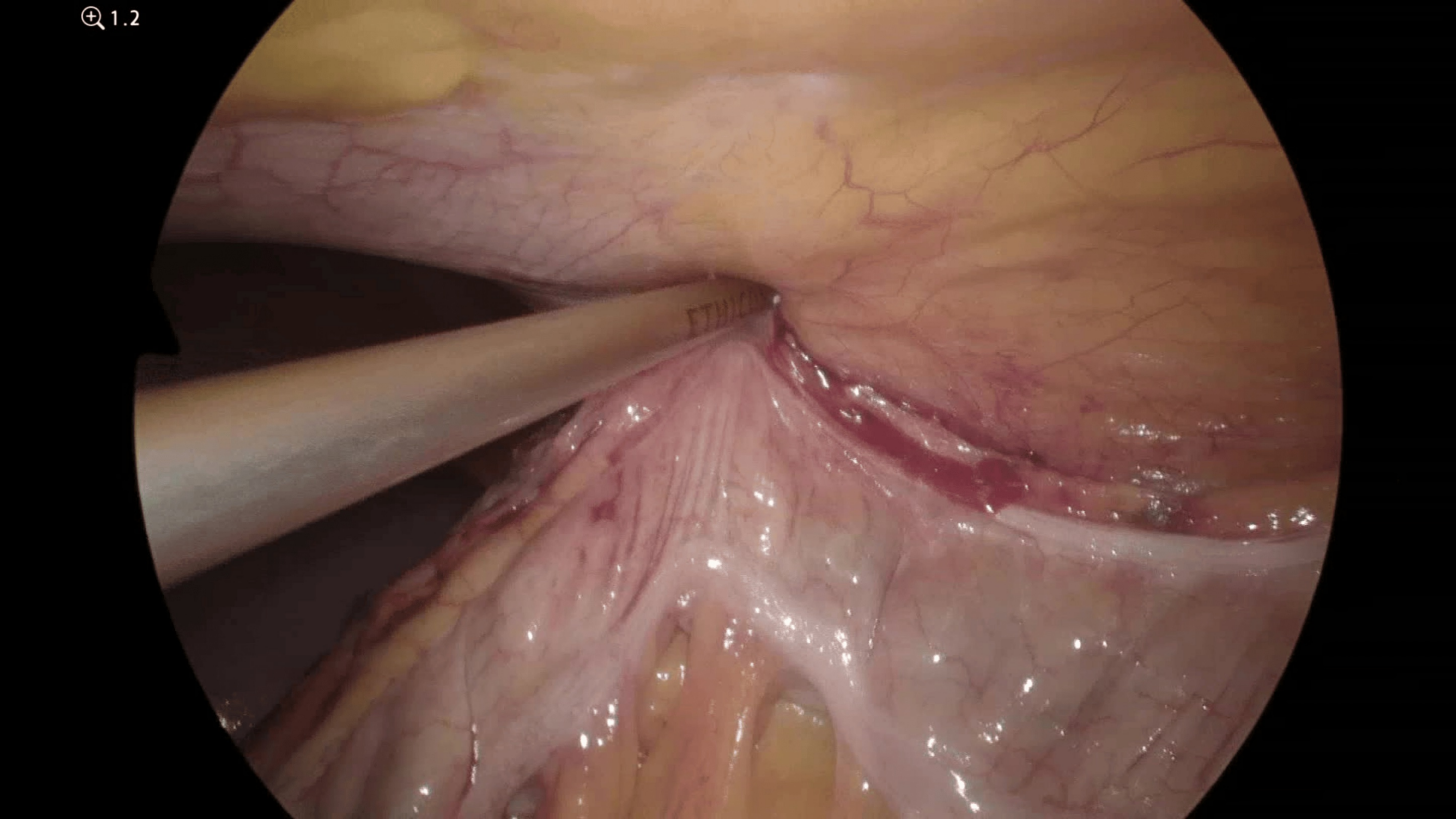
Securing the peritoneum with absorbable tackers. The mesh was covered by the peritoneum, which was then secured in place using absorbable tackers.

Under general anesthesia, the patient was positioned in the right lateral decubitus position. The abdomen was insufflated with carbon dioxide (CO2) to a pressure of 15 mmHg through an incision just left of and superior to the umbilicus. A 5 mm trocar was inserted for the 5 mm scope. A 5 mm and a 12 mm trocar were inserted in the left subcostal and left abdomen, respectively. The left colon was mobilized medially along the line of Toldt toward the splenic flexure using a hook. The lumbar defect was located (Figure 3), and the ilioinguinal nerve was identified and preserved. The defect was debrided of surrounding fat and was measured to be 3 cm in diameter. At this point, the insufflation pressure was reduced to 10 mmHg. The defect was closed (Figure 4) using a non-absorbable 0 running V-Loc suture (Medtronic, Dublin, Ireland). A Symbotex mesh (Medtronic) of a diameter of 10 cm was deployed over the reduced defect and was secured with absorbable tacker to the surrounding muscles (Figure 5). The mesh was covered by the peritoneum using absorbable tackers (Figure 6). Postoperatively, the patient's course was uneventful, and she was discharged on postoperative day one. All patient care was performed at Carmel Medical Center in Haifa, Israel.

## Discussion

While a small number of lumbar hernias are congenital (20%), the majority are acquired (80%). Most acquired lumbar hernias are left-sided and occur in male patients between 50 and 70 years old [5]. Of the acquired lumbar hernias, 55% are spontaneous, and 25% are the result of trauma, surgery, or inflammation. The hernia may contain retroperitoneal fat, kidney, colon, small bowel, omentum, stomach, ovary, spleen, or appendix [6]. As lumbar hernias are very rare, several case reports have been published. Our literature review attempts to summarize common data points between many of those reports.

Our literature review covered 37 case reports (41 patients), a long-term prospective study, and a case study on a novel surgical technique. Of the 41 patients in the case reports, 19 were treated using a laparoscopic approach. Of the 41 patients, 11 had complications following hernia repair (26.8%), the most common being recurrence, which occurred in seven patients (three after a laparoscopic approach, four after open repair). Other complications noted were bowel obstruction (two patients), seroma (two patients), hematoma (one patient), and sepsis (one patient). Of the 11 patients who reported complications, four of them had an open procedure, and eight had a laparoscopy (one underwent both open and laparoscopic procedures and had complications after both procedures). Where data were available, we calculated that the average length of stay for laparoscopic cases was 1.46 days. In patients who underwent an open procedure, the average length of hospitalization was 1.67 days. Our review found that the most common causes of traumatic injury in patients who went on to develop a lumbar hernia were motor vehicle accidents (29.2% of all patients) or a bone graft taken from the iliac crest (14.6%).

No patients were noted to have a surgical history like the patient in our case report (breast implants, implant removal, and abdominoplasty). A 2020 retrospective study on the correlation between improved back pain and abdominoplasty suggested that abdominoplasty may lead to an increase in abdominal pressure and a reduction of anterior abdominal weight and can therefore alter the biomechanics of trunk musculature [7]. Following this line of thinking, there is a possibility that the abdominoplasty may have contributed to the development of a lumbar hernia. However, due to ours being a unique and isolated case, it cannot be proven that the abdominoplasty was the cause of the hernia.

Lumbar hernias are not difficult to diagnose, but they are commonly mistaken for other conditions due to their scarcity. Diagnosis can be made through physical exam and clinical symptoms, but the most definitive way is through computed tomography imaging. Differential diagnoses can include a wide range of conditions, varying greatly in severity: lipoma, abscess, hematoma, sarcoma, renal tumor, and adrenal tumor. Imaging is an important tool in ruling out other conditions, as well as in identifying the location and surroundings of the lumbar hernia before surgery.

Lumbar hernias usually present as a raised flank mass with little to no symptoms. They can increase with exertion or activity and disappear when lying down (prone or supine). Possible symptoms include generalized dull pain or discomfort, abdominal pain, and localized tenderness. Severe symptoms can arise secondary to complications such as strangulation (stemming from hernia incarceration) and bowel obstruction. Bowel incarceration occurs in approximately 25% of patients [6]. These patients may present with vomiting, abdominal pain, and distension [8].

Surgical repair is the recommended treatment due to the risk of hernia incarceration. In the past, large lumbar hernias were reported as being treated with mesh fixation and/or fascial flap via a long skin incision from the 12th rib to the iliac crest [3,9]. While this method has proved effective in repairing the defect, it involves significant retroperitoneal dissection [3].

The initial laparoscopic technique involved a transabdominal approach, anchoring the mesh to the 12th rib superiorly, iliac crest inferiorly, erector spinae fascia medially, and external oblique fascia laterally [3,10].

Tension-free repairs using a retroperitoneal laparoscopic approach have been performed successfully [11]. It can be inferred that the usual benefits of a laparoscopic approach (versus an open one) would offer the same benefits in a lumbar hernia procedure as it does in an inguinal hernia procedure (smaller wound, less pain during recovery, and faster recovery) [12]. However, based on our literature review, laparoscopic repairs showed to have a higher complication rate (19.5%) compared to open repairs (9.7%), with the most common complication being recurrence. While recurrence rates vary depending on multiple factors such as hernia type and surgeon’s expertise [13], a laparoscopic approach in hernia repairs generally leads to a lower recurrence rate [14]. It is possible that, due to lumbar hernias being so rare, a laparoscopic approach may not be the proper one, as most surgeons likely have not encountered one before in their career. Further supporting this point, in two of the seven cases of recurrence (28.5%), an open surgical approach was used for the revision surgery, where the initial repair had been attempted laparoscopically. In practice, recurrence poses further risks to the patient with hernia incarceration, as well as the usual risks that accompany another surgery (infection, bleeding, etc.), so it is important to choose the correct initial surgical approach.

Table 1 summarizes the findings of the literature review on lumbar hernia.

**Table 1:** Findings of the literature review on lumbar hernia. M: male; F: female; COPD: chronic obstructive pulmonary disease.

Case #	References	Repair type (Open/Lap)	Age (years)	Gender (M/F)	Symptoms (pre-repair)	Complications	Length of stay	Link to case report	Hernia site	Trauma history
1	Hide et al., 1999 [7]	Laparoscopic	70 years	F	Large bowel obstruction secondary to incarceration of the mid descending colon - constipation for one week, vomiting, and vague lower abdominal pain.	None	7 days	Link	Inferior	Bilateral hip replacements, appendectomy, 3 cesarian sections
2	Nam et al., 2011 [8]	Laparoscopic	70 years	F	Dull, mild pain in a lump in her upper left posterior back.	None	5 days	Link	Superior	None
3	Heniford et al., 1997 [9]	Laparoscopic first, followed by open	42 years	M	Left-sided mass in the inferior lumbar region.	Recurrence 10 days post-op, second recurrence accompanied by mechanical bowel obstruction and signs of sepsis 14 days later.	Not mentioned	Link	Inferior	Motor vehicle accident
4	Habib, 2003 [11]	Laparoscopic	65 years	M	Right-sided lumbar mass was responsible for pain.	Subcutaneous seroma, drained with needle suction. Diagnosed on postoperative day 12.	2 days	Link	Superior	None
5	Park et al., 2009 [15]	Open	71 years	M	Presented with a mass in the right lumbar region.	Recurrence 6 months post-op	1 day	Link	Grynfeltt hernia (superior)	Surgery a year and a half prior at the same location for a suspected lipoma.
6	Lee et al., 2010 [16]	Open	83 years	F	Gradually increasing mass accompanied by intermittent right abdominal and groin pain.	None	5 days	Link	Superior	None
7	Salameh et al., 2004 [17]	Laparoscopic	64 years	M	Presented with lower back pain and swelling on the left side.	New-onset atrial fibrillation prolonged hospital stay. Hernia recurrence	Not mentioned	Link	Posterior lateral	Right radical nephrectomy 3 years prior. Lumbar hernia 1 year post-op. Repaired but recurred shortly after.
8	Ziesmann et al., 2014 [18]	Laparoscopic	74 years	F	Signs and symptoms of small-bowel obstruction and a clinically appreciable, irreducible, left-sided lumbar hernia associated with previous iliac crest bone graft harvesting.	Upon admission: myocardial infarction + flash pulmonary edema. Post-op: paralytic ileus	7 days	Link	Superior posterior	Bone graft harvesting from the iliac crest.
9	Madan et al., 2006 [19]	Laparoscopic	23 years	M	None	Laparoscopic attempt failed, required an open approach due to extensive adhesions. Hernia recurrence.	1 day	Link	Inferior	Motor vehicle accident - pelvic fracture, bowel resection, open tibia fracture
10	Madan et al., 2006 [19]	Laparoscopic	64 years	F	None	None	1 day	Link	Posterior lateral	Nephrectomy 4 years prior.
11	Ahmed et al., 2014 [20]	Open	45 years	F	Vague lumbar pain and a mass in the right lumbar region.	None	1 day	Link	Superior	None
12	Piozzi et al., 2019 [21]	Open	87 years	F	Slightly tender swelling in the right lumbar area.	None	3 days	Link	Grynfeltt hernia (superior)	Carotid aneurysm embolization, left hip prosthesis insertion.
13	Orcutt et al., 1971 [22]	Open	47 years	M	Complained of a "knot" in his side after straining himself 3-4 weeks earlier. The mass was painful and enlarging.	None	7 days	Link	Superior	Strained himself while loading heavy boxes onto a truck.
14	Roham et al., 2018 [23]	Laparoscopic	47 years	M	Pain at the hernia site 6 months after initial presentation.	None	Not mentioned	Link	Superior	Motor vehicle accident - Non-displaced sternal fracture, intraperitoneal bladder rupture, avulsion of abdominal wall musculature from the iliac crest.
15	Pang et al., 2019 [24]	Open	54 years	M	Presented to ED 2 hours after developing sudden-onset left flank pain and swelling after forceful coughing. Had nausea and vomiting.	None	1 day	Link	Inferior	Stab wound to the inferolateral back 20 years earlier. Radiofrequency ablation of a lumbar facet nerve 1 year prior.
16	Nakanishi et al., 2020 [25]	Laparoscopic	84 years	F	Tennis ball-sized reducible bulge in the mid aspect of the right back.	None	7 days	Link	Superior	Distal gastrectomy and Roux-en-Y reconstruction for gastric cancer.
17	Woodward et al., 1999 [26]	Laparoscopic	71 years	F	1-year history of left flank bulge.	Open attempt failed; hernia recurred 3 months later.	2 days	Link	Inferior	Radiation treatment for thyroid cancer metastasis to the lungs. "A back operation" in which a segment of the left iliac crest bone was grafted.
18	Moreno-Egea et al., 2013 [27]	Surgical options in lumbar hernia: laparoscopic versus open repair. A long-term prospective study	Lap: 61.6 ± 11.6. Open: 64.2 ± 8.6	Laparoscopic: 17 (M), 18 (F). Open: 6 (M), 14 (F)	N/A	Laparoscopic: hematoma: 4; seroma: 7; transitory pain: 2. Open: hematoma: 0; seroma: 8; transitory pain: 0	Laparoscopic: 2.5 ± 0.9	Link	Laparoscopic: superior: 16; inferior: 19. Open: superior: 4; inferior: 9	Laparoscopic: surgery: 25; trauma: 7
							Open: 5.1 ± 1.8			Open: surgery: 15; trauma: 5
19	Matsuhashi et al., 2006 [28]	Open	63 years	F	Sudden epigastric pain, abdominal distention, severe lower abdominal pain, and left back pain. Palpable mass in the left supra-iliac region. Incarcerated defect of the iliac.	None	At least 9 days, total stay not mentioned.	Link	Superior	Luxatio coxae-performed bone graft
20	Mingolla et al., 2009 [29]	Open	40 years	F	Presented with a diagnosis of recurrent lipoma. Palpable mass and mild occasional pain.	None	Discharged the same day	Link	Superior	None
21	Skrekas et al., 2005 [30]	Open	60 years	M	Complaints of swelling in the left lumbar region associated with dull aching pain for 2-3 months.	On day 15 after the operation, a 100-ml flank seroma was evacuated by a simple needle aspiration.	3 days	Link	Superior	None
22	Losanoff et al., 2002 [31]	Open	65 years	M	Sudden onset of pain in the left lumbar area accompanied by nausea and vomiting. Tenderness in the left lumbar area, along with hypoactive bowel sounds.	None	6 days	Link	Inferior	None
23	Astarcioğlu et al., 2003 [32]	Open	70 years	F	3-day history of abdominal pain, distention, and vomiting. Distended abdomen with hyperactive bowel sounds. Tender left flank mass.	None	7 days	Link	Inferior	None
24	Burt et al., 2004 [33]	Open (3 case reports)	-	-	(1) Persistent dull right flank pain 2 months after traumatic injury. (2) Persistent left flank pain 5 months after traumatic injury. (3) Persistent left back pain 5 months following a motor vehicle collision.	Patient #3 presented 6 months later with recurrence.	(1) 2 days. (2) 3 days. (3) 3 days	Link	(1) Inferior	(1) Jumped from a 3-story rooftop. (2) Skiing accident. (3) Motor vehicle accident
									(2) Inferior	
									(3) Not given	
25	Light et al., 2010 [34]	Open	56 years	F	Presented with absolute constipation for one week and lower abdominal pain radiating to the left flank.	None	10 days	Link	Inferior	Para-umbilical hernia repair, anterior vaginal wall repair, and spinal fusion.
26	Patnaik et al., 2015 [35]	Laparoscopic	61 years	M	Presented with 6 months of pain and swelling in the left lumbar region for the last 6 months.	None	3 days	Link	Inferior	None
27	Radais et al., 2011 [36]	Open	76 years	M	Patient was admitted for acute COPD exacerbation. 3 days later, he experienced acute intestinal obstruction.	None	Not mentioned	Link	Transiliac	30 years after iliac bone harvesting for femur surgery.
28	Scheffler et al., 2015 [37]	Laparoscopic	92 years	F	Presented with right-sided abdominal pain, loss of appetite, nausea, and absence of gas and stool for 24 hours. Small bowel obstruction found on imaging.	None	10 days	Link	Superior	Right lumbar scar noted on inspection, no record of previous abdominal surgery.
29	Di Carlo et al., 2007 [2]	Laparoscopic twice, then open	42 years	M	Presented with an inferior lumbar hernia. Upon presenting with recurrence, the patient showed signs of mechanical bowel obstruction.	The hernia recurred 10 days after the initial repair.	Not mentioned	Link	Superior	Motor vehicle accident
30	Rafols et al., 2020 [38]	Laparoscopic	83 years	F	Right flank soft tissue mass that had been progressively increasing in size.	Hematoma that resolved spontaneously	1 day	Link	Superior	Bilateral mastectomy without reconstruction, total hysterectomy and oophorectomy, total colectomy with ileocolic anastomosis for colonic inertia, T12-L2 fusion, contralateral left-sided open lumbar hernia repair with mesh 2 years prior.
31	Grauls et al., 2004 [39]	Laparoscopic	83 years	F	Patient presented with lower back pain and swelling on the left side.	Unknown	Unknown	Link	Superior	Unknown
32	Alcoforado et al., 2013 [40]	Open (3 case reports)	52-75 years (3 men)	M	All three complained of pain and a tumor in the lumbar region. None had a history of trauma or surgery.	None	Same day	Link	Superior	None
33	Meinke, 2003 [41]	Laparoscopic	78 years	M	Intermittent, dull right groin and thigh pain.	None	5 days	Link	Inferior	Lithotripsy 1 year earlier and right iliac wing bone harvest 3 years earlier.
34	Ipek et al., 2005 [42]	Laparoscopic	36 years	F	Chronic left lumbar pain and a sensation of a growing mass.	None	1 day	Link	Inferior	Small bowel resection after a motor vehicle accident 8 years prior, laparoscopic cholecystectomy 6 years prior.
35	Saboo et al., 2014 [43]	Laparoscopic	22 years	M	Presented with severe abdominal pain following a fall from a motorcycle onto his right side. Crepitus and ecchymosis over the right flank were present.	None	2 days	Link	Inferior	Motorcycle crash
36	Jarrahy et al., 2003 [44]	Open	37 years	M	Progressively worsening flank pain.	None	Not mentioned	Link	Inferior	High-speed rollover motor vehicle accident 2 weeks prior.
37	Do et al., 2012 [45]	Open	61 years	F	Presented with a week-long history of painful swelling along her right flank.	None	Not mentioned	Link	Superior	A year and a half prior, underwent Iliac crest bone graft harvesting to repair an odontoid fracture after a motor vehicle accident.
38	Sarwal et al., 2019 [46]	Laparoscopic	65 years	F	Abdominal discomfort and swelling on the left flank for 2 years	None	2 days	Link	Superior	None
39	Carbonell et al., 2005 [47]	Open (not a case report)	Mean age was 50 years. Range was 31-78	6 men, 4 women	Novel retro-muscular lumbar hernia repair technique - five hernias were recurrent, five were incarcerated, seven were incisional hernias, and three were posttraumatic. Back and abdominal pain were the most common presenting symptoms.	None	Mean length of stay was 5.2 days	Link	N/A	Three patients had posttraumatic hernias following car accidents.
40	Sethi et al., 2025 [48]	Laparoscopic	57	M	None	Open	Not mentioned	Link	Superior	None

## Conclusions

Lumbar hernias are rare and typically present as a palpable flank mass that may be initially mistaken for a lipoma or other soft-tissue growth, as occurred in our case. Physicians should maintain a high index of suspicion for lumbar hernia in patients with reducible flank masses, particularly those with a history of trauma such as motor vehicle accidents or previous iliac crest bone grafting. Although it cannot be proven, it is possible that our patient’s prior abdominoplasty contributed to hernia development. Our literature review indicates that laparoscopic repair is associated with a shorter hospital stay; however, recurrence remains a significant concern (17%), likely due to the anatomical complexity of lumbar hernias and limited surgical experience. Ultimately, the surgical approach should be individualized according to hernia site, surgeon expertise, and shared decision-making with the patient to optimize outcomes and minimize recurrence risk.
